# Learned Irrelevance, Perseveration, and Cognitive Aging: A Cross-Sectional Study of Cognitively Unimpaired Older Adults

**DOI:** 10.3390/brainsci13030473

**Published:** 2023-03-10

**Authors:** Aleksandra Fijałkiewicz, Krzysztof Batko, Aleksandra Gruszka

**Affiliations:** 1 Doctoral School in the Social Sciences, Jagiellonian University, 30-010 Cracow, Poland; 2 Institute of Psychology, Jagiellonian University, 30-060 Cracow, Poland; 3 Department of Research and Design, Medicine Economy Law Society (MELS) Foundation, 30-040 Cracow, Poland

**Keywords:** learned irrelevance, cognitive flexibility, attentional set-shifting, cognitive aging

## Abstract

The effect of natural aging on physiologic mechanisms that regulate attentional set-shifting represents an area of high interest in the study of cognitive function. In visual discrimination learning, reward contingency changes in categorization tasks impact individual performance, which is constrained by attention-shifting costs. Perseveration (PE) and learned irrelevance (LI) are viewed as two different mechanisms that shape responses to stimuli, which are predicated on the shift in stimulus form. To date, only studies examining patients with Parkinson’s disease have provided some insight into the relationship between individual age and performance in PE and LI tasks. We enrolled 60 healthy individuals (mean [SD] age, 63.0 [12.6]) without a history of dementia, a cerebrovascular incident, or a neurodegenerative disease. No association was observed between crystallized intelligence or verbal fluency scores and reaction time in both PE (r = 0.074, *p* = 0.603; r = −0.124, *p* = 0.346) and LI (r = −0.076, *p* = 0.562; r = −0.081, *p* = 0.536) task conditions, respectively. In contrast, a statistically significant linear relationship was observed between age and reaction time (RT) for PE (r = 0.259, *p* = 0.046) but not for LI (r = 0.226, *p* = 0.083). No significant linear relationship was observed for changing RTs in PE and LI (r = 0.209, *p* = 0.110). The present study is the first report that provides a descriptive overview of age-related differences in PE and LI in a sample of cognitively unimpaired middle- to older-aged adults.

## 1. Introduction

The term “attentional set-shifting” refers to the human ability to function effectively under changing environmental conditions. Shifting attention refers to the transfer of attention from one stimulus or dimension to an alternative one. This action carries a cognitive cost and can be quantified by individual reaction time and error rate. However, several mechanisms are thought to underlie attentional set-shifting, of which perseveration (PE) and learned irrelevance (LI) are most known. In visual discrimination tasks, PE refers to the difficulty in shifting attention away from previously relevant information, while LI is the inability to shift attention to previously irrelevant stimuli [1]. Although similar at first glance, LI and PE are considered to be independent phenomena on a behavioral, neurochemical, and neuroanatomical level [1,2,3,4,5]. While PE has been associated with frontal lobe function and dopamine dependence [1,6,7], the mechanisms underlying LI remain unclear. Although the negative effect of aging on PE has been reported consistently [8,9,10], little is known about how age affects set-shifting in LI. With the increasing proportion of elderly in the general population, understanding how this process affects attention is of high interest.

Out of the two phenomena, PE has been described to a greater extent. It has been proposed that older individuals are more likely to repeat the same strategy for subsequent problems, even if such a strategy is no longer appropriate [8,9,10,11,12]. Studies also indicate a relationship between neurodegenerative changes (especially in frontal areas) and an increased tendency toward perseverative reactions [9]. Comparing elderly individuals with patients exhibiting frontal lobe dysfunction often reveals a similar mechanism of perseverative errors in both groups. Frontal functions are attributed a critical role in modulating perseverative responses [8,10,13].

When utilizing frontal function-sensitive tests, such as the Wisconsin Card Sorting Test (WCST), lower scores and a greater rate of perseverative reactions have been observed in older individuals compared to younger ones [8,10]. In the WCST, achieving new categories requires a change of mental setting. This reflects overcoming a pattern that has become irrelevant and implementing an appropriate new scheme [10,14]. An individual’s propensity for this phenomenon determines performance in task-switching (i.e., switch cost) [15]. Similar to patients with frontal lobe deficits, older individuals have difficulties learning and applying WCST’s rules, as is reflected by a higher number of perseverative errors and worse performance. The age-related increase in perseverative errors is also observed in fluency tests (both verbal and non-verbal). A significant increase in perseveration error rates has been observed after the age of forty [9,16].

Unlike PE, the LI phenomenon remains a challenge for researchers. It was first discovered by Mackintosh (1973) [17], who studied animal learning. The occurrence of LI in humans was described by Owen et al. (1993), whose work was conceptualized around attentional set-shifting in patients with Parkinson’s disease (PD) or in cases with frontal lobe excision. This study by Owen et al. (1993) was conducted in the intra- and extra-dimensional (IDS/EDS) visual learning paradigm, with LI and PE treated as independent conditions. The paradigm was modeled after the classic WCST but allowed for a more accurate study of basic cognitive functions [18].

In IDS, participants shift attention to a new exemplar of the same dimension (e.g., a color change from green to red). In contrast, in EDS, participants shift attention to a new, previously irrelevant stimulus dimension (e.g., from color to shape). Difficulties in EDS shifting may stem from both LI and PE. Owen et al. (1993) observed that patients with frontal lobe excisions had a higher error rate in the PE condition in comparison to healthy individuals and PD patients. However, performance in the LI condition was comparable to that of healthy controls. In contrast, non-medicated PD patients exhibited low performance in both the PE and LI conditions, while medicated patients were impaired only in the LI condition. The results described by Owen et al. (1993) improved our understanding of the nature of attentional set-shifting deficits: (i) both PE and LI likely contribute to the cognitive impairments observed in PD, see also [19]; (ii) PE but not LI is a purported determinant of set-shifting impairment in frontal lobe patients. Set-shifting impairments in PD and frontal lobe patients likely involve fundamentally different, though related, cognitive processes. Interestingly, treatment with L-dopa does not appear to improve performance in the LI condition, which may reflect a dopamine-independent regulatory mechanism [1,5,20].

As of yet, no studies have investigated the effect of age on LI directly, which contrasts with abundant data on cognitive aging in PE, e.g., [8,9,10]. So far, only one behavioral study utilizing the visual discrimination learning paradigm, conducted by Slabosz et al. (2006), implicated no age-related effect. However, this research did not intend to study aging effects on LI, but to determine the effect of dopamine on LI in PD patients. Thus, the study included two control groups of older and younger individuals, treating age as a categorical variable (the drawbacks of such an approach have been described elsewhere [21]). As a result, the study revealed no significant differences across the two control groups. However, conclusions drawn from an extreme group design must be treated cautiously. It can be argued that such results represent a special case of oversampling, in which the external parts of the distribution are sampled but the central part is completely excluded [22]. Furthermore, Slabosz et al. (2006) used the IDS/EDS visual discrimination learning paradigm optimized for examining specific populations, e.g., neurological patients (frontal lobe deficits or PD) [1,5,19,23]. Consequently, it remains unclear whether the same paradigm is sensitive enough to detect differences in normal aging populations. It is also difficult and potentially unreliable to generalize results from specific case studies to the general population.

Studies based on the IDS/EDS paradigm also suffer from a confounding effect of novelty; for details, see [5]. When a novel dimension is introduced at the EDS stage, individuals must choose between the previously irrelevant dimension and the novel one. Several investigators have pointed out that the response to novelty involves specific potentially confounding mechanisms and may even account for the LI phenomenon [5,24]. Indeed, the involvement of working memory could introduce an additional degree of uncertainty [25,26].

The necessity to “work out” the correct response in the visual discrimination learning paradigm represents another concern; see [27]. In a typical task, participants are unaware of which stimulus they should respond to and have to choose the correct response in a trial-and-error process. They undertake a decision-making process involving stimulus recognition (i.e., discrimination between two stimuli), goal ascertainment, and stimulus choice (i.e., in relevance to the task target). Therefore, task performance depends not only on the studied phenomena but also on other functions. Thus, poor performance could be attributed to deficits in visuospatial learning, speeded processing, working memory, and set formation [28,29]. Hence, to counter the contaminating effects of other mechanisms, it may be advantageous to use an explicit cue, such as a clear instruction regarding which stimulus the participants should respond to. Having participants switch predictably may reduce requirements on learning and working memory load [15,29,30].

To overcome the methodological limitations of the previous attempts to evaluate the effect of physiological aging on the LI phenomenon, in the current study, we decided to use an alternative to the IDS/EDS approach as proposed by Dreisbach and Goschke (2004) [31]. This paradigm is fundamentally based on switching and learning. It resembles the task-switching paradigm [32], in which individuals respond to target stimuli that appear in a predetermined color while ignoring distracter stimuli in a different color; this is followed by task transfer to one of two switching conditions. In the PE condition, participants respond to stimuli in a new color, while distractors appear in the previous target color. For LI, participants respond to stimuli in the previously ignored color, while distractors appear in a new color.

The approach proposed by Dreisbach and Goschke (2004) has been validated in studies of individual differences in cognitive flexibility in the general population [31,33,34,35,36]. It allows for comparing the effects of age on both LI and PE in the same paradigm (in contrast to the study by Slabosz et al. (2006), which focused solely on LI). Furthermore, we recruited a wider age range of individuals (40–80 years) as the age-related decline in cognitive flexibility is not observed until middle age [37]. Lastly, we decided to treat age as a continuous variable. Due to this modified research plan, we want to better understand the LI phenomenon and its relationship with cognitive aging. Regarding the study by Słabosz et al. (2006), we hypothesize that age affects PE but not LI.

## 2. Methods

### 2.1. Participants

This study enrolled 60 otherwise healthy individuals at or over the age of 40 (mean age = 63.0; SD = 12.6, 46 female). Individuals were not eligible for the study if they (1) did not provide written informed consent or (2) had a documented diagnosis of neurological or psychiatric illness, as reported by the participants themselves or within their available medical records. Recruitment was performed using a convenience sample from senior clubs and community volunteers. These two different recruitment pools were necessary in order to achieve a sample with a wide range of late adulthood. Participants were also queried regarding a history of dementia or a cerebrovascular incident, both of which precluded them from inclusion in the study. Individuals over 60 years of age were further tested with the MoCA test, where the cut-off threshold for inclusion was 24 points. Power calculation revealed that for a small to modest effect (defined as r = 0.20 to 0.39), with alpha set at 0.05 and power at 0.80, we would need to recruit between 40 and 150 participants (approximately).

### 2.2. Outcome Measures

To accurately measure reaction time and accuracy in the LI and PE conditions, we adopted a previously described and validated task by Dreisbach and Goschke (2004). Co-variates of theoretical interest were pre-defined as crystallized intelligence, as measured by a vocabulary knowledge test [38] and verbal fluency, as reflected by a verbal fluency task [39].

### 2.3. Materials and Procedure

1. The verbal fluency task [39] is used to assess the ability to retrieve and pronounce words fluently according to a set criterion. In this task, the participant lists as many words from a given category as possible within 1 min. There are three categories: animals, words starting with “K”, and sharp objects. The result of the test is the number of words mentioned (separately for each category and the sum of all categories). The internal reliability of this test was assessed using the ωh coefficient at 0.66;

2. The vocabulary knowledge test [38] is a measure of crystallized intelligence. From four given words, participants choose a word that is synonymous with the given word. This test consists of 40 single-choice questions and lasts 5 min. The test result is the sum of the correct answers given. The absolute stability corrected for guesswork for this test was r = 0.89;

3. The attentional set-shifting task was designed by Dreisbach and Goschke (2004) to investigate individual differences in cognitive flexibility vs. stability. The task consists of 4 blocks with 60 attempts in each. In each trial, two stimuli of different colors are displayed simultaneously on the screen, arranged vertically. They are either two letters (A, E, O, U, K, M, R, or S) or two numbers (2, 3, 4, 5, 6, 7, 8, or 9). Letters can be red, blue, or yellow, while numbers can be olive, purple, or gray. Participants are instructed to respond to a stimulus that appears in a predetermined color while ignoring a stimulus of a different color. The location of the up-down stimulus is determined randomly. In individual blocks of trials, participants perform tasks of categorizing whether the letter in the target color is a consonant or a vowel or whether the number in the target color is even or odd, respectively. Participants press the left arrow on the computer keyboard if the stimulus is a consonant or an even number, and the right arrow if the stimulus is a vowel or an odd number. The individuals receive feedback only when they make a mistake.

Target stimuli and distractors may be either compatible (e.g., both stimuli are even numbers, thus requiring the same response, i.e., pressing the same arrow on the keyboard) or incompatible (e.g., the target stimulus is a consonant and the distractor a vowel, thus the correct response is pressing a different arrow on the keyboard than the distractor). Compatible and incompatible conditions appear randomly with two constraints. First, the target stimulus and the distractor are never identical, making incompatible trials more frequent. Second, the first target stimulus after changing the target stimulus is always incompatible [31].

**Figure 1:**
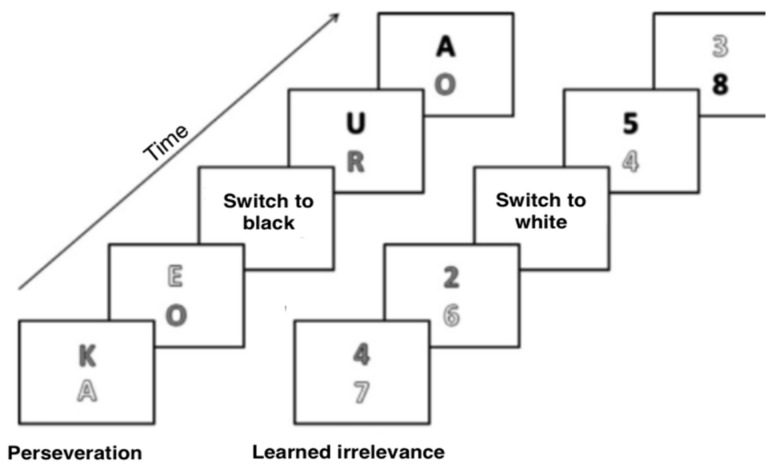
Sample order of trials for PE and LI conditions (the version of the task with letters and the version of the task with numbers are shown). The figure shows two trials before a color change and two trials after a color change. The different colors that appear in the quest are shown here as black, white, and gray. In both examples, gray is the target color before the change. In the LI condition, the target color changes to white, which was previously a distractor. In the PE condition, the previous target color (gray) becomes a distractor, and the new color (here black) becomes the target color. Own elaboration based on Dreisbach and Goschke (2004).

Each block consists of 60 trials. After 40 trials, an indication of a change of color appears. Critical comparisons concern the two intervals immediately before the target color switch (Trials 36–40) and immediately after the switch (Trials 41–46). In LI, the individuals should react to the color that was previously a distractor while the distractor appears in a new (previously absent) color. Under PE, individuals should respond to a stimulus that is a new color (not present before), while the target stimulus from the previous trial becomes a distractor (see Figure 1). For example, if in a given trial the target color is blue and the distractor is red, then in the LI condition the target color will be red and the distractor will be yellow in the next trial. However, in the PE condition, the target color will be yellow and the distractor blue. The tasks (categorizing letters vs. numbers) do not change within one block [31].

The task consists of three blocks in the PE condition and three in the LI condition. The type of task (categorization of letters vs. numbers) and condition (PE vs. LI) change with each block. The order of the conditions is balanced. The respondents are instructed to answer as quickly as possible while avoiding mistakes. The task starts with 20 training trials.

To conclude, the following factors are present in the task: 2 types of attention shifting (condition: PE vs. LI) × 2 types of blocks (condition: before vs. after change) × 2 levels of compatibility (compatible vs. incompatible condition). Dependent variables: reaction time (RT) and accuracy. The internal reliability of this test was assessed using the ωh coefficient, which was 0.89.

For statistical analysis, the percentage change in RT or accuracy was utilized as an outcome measure (e.g., the quotient of the condition prior to and after the stimulus change). We refer to these variables as RT and accuracy. Only variables in the incompatible condition were included in the analysis. For RT, only trials in which the correct answer was given were analyzed. Analysis was performed in R 4.2.2 (R Core Team, 2022). Categorical variables are summarized using counts and percentages, while continuous variables are reported as median and interquartile range (IQR) or mean and standard deviation (SD). Pearson’s correlation was calculated for pairs of continuous variables or Spearman’s rho if a monotonic but nonlinear relationship was suspected. Ordinary least-squares regression models were constructed for multivariable analyses, and variables were selected using the stepwise format and the AIC criterion. Tests were two-tailed, and a *p*-value was considered significant at <0.05.

## 3. Results

The study sample included middle- to older-aged individuals, of which the majority were female and had an average performance in intelligence and verbal fluency scores (for details, see Table 1).

**Figure 2:**
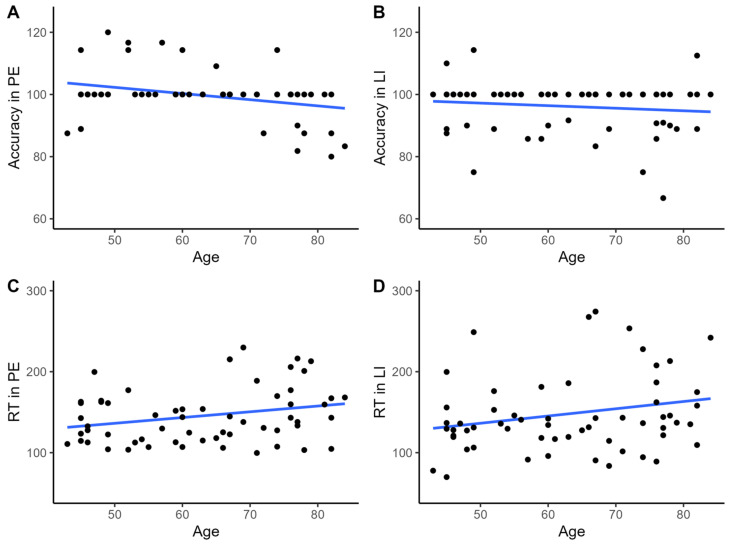
A scatter plot with the line of best fit is used to illustrate the relationship between age and accuracy rates (**A**,**B**) or reaction times (**C**,**D**) in apparently healthy individuals.

A statistically significant linear relationship was observed between age and RT for PE (r = 0.259, *p* = 0.046), in contrast to LI (r = 0.226, *p* = 0.083, see Figure 2C,D). No monotonic association (see Figure 2A,B) was observed for task accuracy in the LI condition (rho = −0.108, *p* = 0.410) in contrast to PE (rho = −0.287, *p* = 0.026).

**Figure 3:**
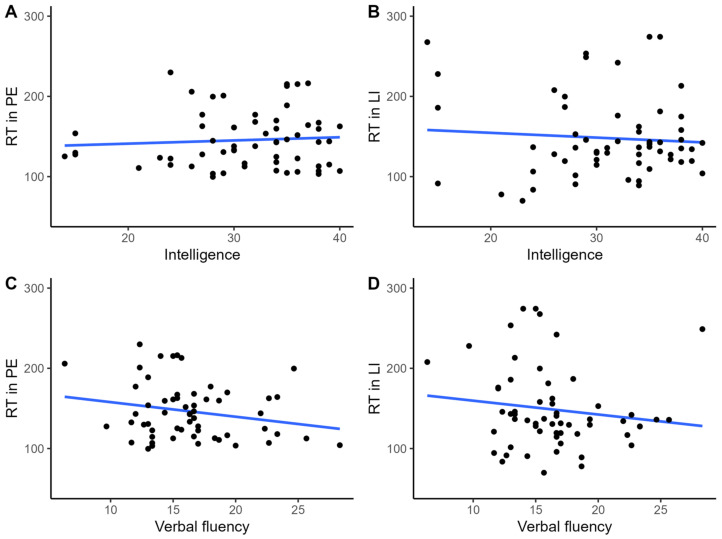
A scatter plot with a line of best fit to illustrate the relationship between intelligence score in Choynowski’s test and verbal fluency test with reaction times in the PE (**A**,**C**) and LI (**B**,**D**) conditions measured in apparently healthy individuals.

No statistically significant linear relationship was observed between intelligence or verbal fluency scores and RTs in both the PE (r = 0.074, *p* = 0.603; r = −0.124, *p* = 0.346) and the LI (r = −0.076, *p* = 0.562; r = −0.081, *p* = 0.536) conditions, respectively (see Figure 3).

**Figure 4:**
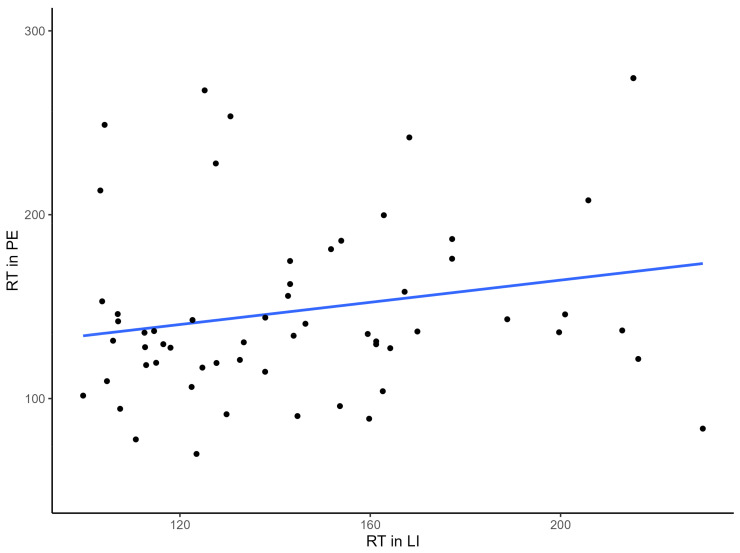
A scatter plot and linear fit illustrating the relationship between reaction time in the LI and PE conditions.

There was also no significant linear relationship between the changing RT in PE and LI (see Figure 4, r = 0.209, *p* = 0.110).

Full multivariable models (i.e., including all co-variates of theoretical relevance studied at present) were constructed for reaction times in both LI and PE conditions (see Table 2). Stepwise selection using the Akaike information criterion was utilized to determine parsimonious models and select predictors of interest. The final model for PE retained only age, while both age and gender were retained in the final model for LI. Additionally, we constructed confidence intervals for the R2 values of both models.

## 4. Discussion

The present study examines the relationship between age and performance in an attention set shifting task measuring PE and LI. In a sample of healthy middle- to older-aged individuals, age was shown to be a statistically significant predictor of performance in PE but not in LI. This is the first report that supports the concept of LI as an age-independent phenomenon in healthy individuals, as was implicated previously by Slabosz et al. (2006) on the basis of an exploratory analysis in PD patients [1,5]. The treatment of age as a continuous variable allowed for a more comprehensive overview of the nature of this relationship. Furthermore, performance was evaluated as a percentage change to account for baseline individual differences. The present study assumed that reaction time is a manifestation of set-shifting costs, thus extending the previous approach. Slabosz et al. (2006) had to focus on the error count due to the clinical manifestations of PD, which is characterized by heterogenous manifestations [40,41], which can be viewed as a confounding variable. Another advantage of this study is the validation of the outcome measure in prior studies on individual differences [31,33,34,35,36].

There is an array of evidence that suggests performance in PE declines with age, but whether this cognitive decline affects LI remains uncertain. Our results are consistent with other data suggesting that some mechanisms (within shifting abilities) responsible for proper cognitive function deteriorate with age while others do not [37,42,43,44]. Others have conceptualized a rate of decline for cognitive ability [45,46], which is a more sensible approach in our view. The decline in attentional set-shifting should be regarded on an individual level within a continuum of age.

Set shifting has consistently been linked with frontal structures and subcortical basal ganglia function [47,48,49]. While the anatomical basis of LI remains unclear, PE is categorized as a frontal deficit. The decline in prefrontal performance and the severity of PE appear as a physiological aspect of age-related cognitive decline [8,9,10,11,12]. Conversely, the lack of or slower deterioration of LI function with age supports the hypothesis that this function is not related to the frontal area. It can be suspected that the LI and PE phenomena are independent of each other, although a research gap remains on a mechanistic, neuroanatomical level. The work by Gruszka et al. (2010) first showed that the LI condition activates the anterior cingulate cortex; in turn, overcoming LI was tied to stimulation of the caudate nucleus, which is a component of the basal ganglia and is also frequently associated with attentional set shifting and general executive function [47,48,49,50]. Moreover, the caudate nucleus is a structure that does not degenerate with age [51,52,53]. The role of the basal ganglia is supported by data from research into PD. This population is of interest as an extreme group comparison. The severity of LI may be a PD-specific symptom that is likely unrelated to a dopaminergic deficit [1,5]. It has been shown that after a dopaminergic drug switch, patients with PD begin to handle PE errors better, while LI errors do not improve. This falls in line with the Dual Syndrome Hypothesis [54], which provides an explanation for the clinical heterogeneity observed in PD [55]. Specifically, it is suspected that two separate mechanisms tied to dopaminergic and cholinergic activity affect the manifestations of cognitive disorders in PD. In comparison to dopamine-related pathways, cholinergic alterations are less understood. Observations that LI is a dopamine-independent phenomenon provide an important premise for future study.

In this study, we examined participants with validated tools that are designed to measure crystallized intelligence, verbal fluency, and cognitive impairment. Both crystallized intelligence and verbal fluency are assumed to be unrelated to the aging process. Prior work demonstrates that alterations in crystallized intelligence are somewhat resistant to aging [56,57], and age has a negligible effect on verbal fluency measures [58,59,60]. We did not observe any significant relationship between crystallized intelligence and age, but also for performance in LI and PE. This is consistent with the results of other studies [61]. Conversely, it is surprising that we did not observe a relationship with verbal fluency scores. Previous studies have suggested that the severity of PE is tied to poor performance in verbal fluency tasks [9,62]. We hypothesize that if such a relationship exists, its strength is likely modest, but it is difficult to appropriately evaluate this relationship. Verbal fluency consists of two different components: clustering (i.e., the production of words within subcategories) and switching (i.e., the ability to shift between clusters) [63], of which only clustering was measured in this study. It has been suggested that clustering reflects temporal lobe functions of categorization, while switching requires frontal functions of shifting and cognitive flexibility [63,64]. It is possible that the task used in this study, as used to test cognitive flexibility [31], would be more strongly associated with the switch indicator.

In the light of the current results, the potential moderating effect of gender on the LI phenomenon should be considered. Although a direct or interactive effect was not observed in this study, gender was retained in the parsimonious model for LI performance. This cannot be interpreted as a causal effect. However, due to a distinct neurochemical and behavioral framework that underlies similar phenomena [65,66,67,68], such an association is plausible. To date, only one study by Muller et al. (2007) has described the relationship between gender and the PE and LI phenomena as measured by Dreisbach and Goschke’s (2004) task. The authors did not make any directional hypotheses due to a lack of data justifying such an assumption. Their findings indicate greater cognitive flexibility among men and, thus, a lower PE error rate. This result is in line with other studies [65,69] that imply greater cognitive flexibility to novel stimuli among men. In turn, in the LI condition, women are characterized by slightly better performance. However, according to the authors of that study, this result is difficult to interpret unequivocally due to a heterogenous study sample with considerable gender imbalance [35].

The present report is difficult to compare with other studies as the measures of performance in the studied task conditions are variable. According to the theory of discrimination learning [70], LI (and attentional set-shifting in general) requires extra-dimensional shifts, which refer to reallocating attention to another stimulus feature, e.g., from color to shape. In the paradigm proposed by Dreisbach and Goschke (2004), attention is shifted within one dimension of the stimulus, i.e., within color (e.g., from blue to red). This is more similar to other forms of discriminatory learning (e.g., intra-dimensional shifts or reversal shifting performed due to reward-contingency change) [2,28]. If we adhere to the train of thought described by the pioneer of the LI phenomenon [70], Dreisbach and Goschke’s (2004) task may not involve attentional shifts but might require the overcoming of prepotent responses [71].

The majority of participants obtained very low error rates in both task conditions in our study. This has several implications for the interpretation of the results. Firstly, we utilized a tool designed to measure a specific mechanism. Ideally, the task should be designed to minimize the involvement of other pathways, such as those involved in more complex problem-solving. Due to a ceiling effect, low variability across individuals is observed, which reduces our ability to evaluate the relationship. Similar observations were noted by Dreisbach and Goschke (2004), who also reported low error rates and no association with the tested task condition [31,35]. Another issue worth considering is that, as reported in Figure 2, some participants improved their accuracy level after the change in the target stimulus. We believe this may be due to the following factors. The task by Dreisbach and Goschke (2004) used in this study is based on a learning process. By nature, learning is characterized by high variability between participants. While performing this task, participants repeat the activity many times, and, additionally, before the appearance of each stimulus, they have a cue informing them which stimulus they should respond to. In our opinion, this is what promotes improved accuracy after changing the stimulus. To reiterate, the interference observed in younger participants is weaker than in older individuals. Combined with repeated repetition of the same activity and the appearance of a cue informing about which stimulus to respond to, this may lead to the fact that some individuals can respond even better after the change of stimulus than before.

Several limitations need to be addressed. This study may be underpowered to detect a significant relationship between age and change in reaction time in the LI condition if the effect size is truly small. We are also unable to reliably test for the presence and significance of interactions with gender. Furthermore, p-values for the univariable relationship between age and reaction time in PE and LI conditions could also be viewed as relatively similar (i.e., if considered as a continuous measure of evidence against the null). Taken together, there is a wide degree of uncertainty regarding the conclusions drawn at present, which are tentative on consistent replication of these findings in other samples. Nevertheless, as the first investigation of LI in healthy aging individuals, this study yields several findings that are worthy of consideration. The confidence bounds for the reaction time model estimate in both task conditions show that a large proportion of variability is unexplained by the individual features studied at present. Further, despite the wide age range studied at present, the age-related effect could actually be very small. Therefore, future studies should recruit large samples and include a wide variety of measures for different inter-individual characteristics, which may facilitate the development of a model with satisfactory explanatory value.

In summary, the physiology of cognitive aging is a process of high interest, as understanding the factors that drive its decline may facilitate developing tailored interventions. Cognitive deficits are highly variable across individuals, and aging itself should not be treated as a major determinant of poor performance under different attentional set-shifting conditions. This case study of healthy individuals supplements the current literature (mainly focused on performance in pathology, e.g., PD and schizophrenia) regarding attentional set-shifting. As these processes are crucial for maintaining workability and an adequate quality of life, e.g., [72,73], explaining the explanatory factors for the decline in individual performance is of public interest. The results of the present study provide several implications for designing future studies. Moreover, they show that some cognitive deficits do not substantially decline with age. Whether this may allow clinicians to differentiate healthy aging from its pathological form is a question for the future. However, assessing performance in LI and PE conditions may be relevant for studies on the progression of cognitive decline in PD and its specific manifestations, such as PD-related dementia.

## 5. Conclusions

This is the first study to directly provide a thorough assessment of LI and PE set-shifting in healthy individuals across the middle and older age spectrum. Our results may suggest that LI is an age-independent phenomenon. Conversely, older age is associated with a deterioration in PE performance, which is in line with what was suspected in earlier reports [5]. This report is an exploratory study that provides insights into a research gap in the study of attentional set-shifting in humans. However, due to the limitations of this study, further confirmatory work is necessary.

## Figures and Tables

**Table 1:** Summary statistics for the total sample (N = 60).

Variable	Mean (SD) or N, %
Age, years	63.0 (12.6)
Sex, male	16, 26.7%
Accuracy LI	96.2 (8.37)
Accuracy PE	99.7 (8.06)
Reaction time in LI	148.0 (50.0)
Reaction time in PE	146.0 (34.5)
Intelligence score	31.1 (6.41)
Verbal fluency score	16.1 (4.52)

**Table 2:** Summary of multivariable linear regression models to predict the rate of change in reaction time after the incompatible stimulus. Model selection was performed using stepwise selection with the Akaike information criterion. 95% confidence intervals (CI) for R2 values were calculated using a bootstrap with 10,000 replicates.

Variable	Coefficient (±Standard Error)			
**Task Condition**	**Learned Irrelevance**	***p*-Value**	**Perseveration**	***p*-Value**
Intercept	80.30 (33.16)	0.019	100.74 (22.34)	<0.001
Age, years	0.83 (0.50)	0.105	0.71 (0.35)	0.046
Sex, male	20.97 (14.24)	0.147	-	-
R-squared (95% CI)	0.086 (0.02–0.24)		0.067 (0.02–0.21)	

## Data Availability

Data is available from the authors upon reasonable request.
